# Pathophysiological implications of ventriculoarterial coupling in septic shock

**DOI:** 10.1186/s40635-023-00573-9

**Published:** 2023-12-07

**Authors:** Michael R. Pinsky, Fabio Guarracino

**Affiliations:** 1 Department of Critical Care Medicine, University of Pittsburgh, Pittsburgh, PA USA; 2 Department of Anesthesia and Critical Care Medicine, Azienda Ospedaliero Universitaria Pisana, Via Paradisa 2, 56123 Pisa, Italy

**Keywords:** Arterial elastance, Ventricular elastance, Coupling, Sepsis, Septic shock, Resuscitation

## Take-home message

Proper ventriculo-arterial coupling results in optimal cardiac output, adequate peripheral organ perfusion, and efficient circulation.

The relation between stroke volume and the developed arterial pressure defined by the relative elastances of the left ventricle and arterial circuit reflect ventriculo-arterial coupling. Decoupling of either or both commonly occur in sepsis and are associated with inefficient LV ejection that can lead to LV failure. Ventriculo-arterial decoupling in sepsis can lead to decreased effectiveness of cardiovascular treatments.

## Introduction

The left ventricular (LV) ejection phase interaction between the cardiac function and the arterial system is called ventriculo-arterial coupling (VAC) and reflects global cardiovascular performance and efficiency [1]. The goal of LV ejection is to increase central arterial pressure, such that the organs downstream can autoregulate their inflow of blood relative to their metabolic needs. The determinants of VAC linkage are complex, but beat-to-beat VAC analysis can be assessed at the bedside and help define cardiovascular status and predict responses to therapies. Similar VAC occurs for the right ventricle (RV), but is usually not clinically relevant, because pulmonary arterial load is low. However, if pulmonary arterial pressure rise RV VAC would also become clinically relevant.

Septic shock is a life-threatening condition resulting from an uncontrolled host inflammatory response to infection [2] leading to acute cardiovascular decompensation and severe hemodynamic impairment. A primary feature of septic shock is systemic arterial hypotension owing to vasoplegia, which itself impairs peripheral perfusion, leading to inadequate tissue oxygen delivery independent of total blood flow. The associated intracellular metabolic disturbances result in profound microcirculatory disturbances and multi-organ dysfunction [4].

In this regard, septic shock can disrupt the normal interaction between both the heart ejecting its stroke volume and the arterial central vessels receiving it causing ventricular–arterial decoupling. Such decoupling in septic shock is a key factor in the pathologic processes of hemodynamic instability [1] and the main determinant of cardiovascular responsiveness to specific therapies [3].

This review will focus on the macro-physiological aspects of LV VAC in septic shock and why treatments aimed at restoring cardiovascular homeostasis are affected.

### What is ventriculo-arterial coupling (VAC)

Ventriculo-arterial coupling (VAC) refers to the dynamic interaction between the ventricular pump ejection and the subsequent change in arterial pressure [3, 5–7]. It reflects global cardiovascular performance and efficiency. As such VAC describes the relationship between the contractile function of the ventricles (the left and right ventricles) and the arterial load during each cardiac cycle. As such changes in VAC, either due to disease, time or treatments may trend in ways independent of microcirculatory blood flow and end-organ function, as often do all macrocirculatory measures during shock resuscitation. The left ventricle is responsible for pumping oxygenated blood into the systemic circulation, while the right ventricle pumps deoxygenated blood into the pulmonary circulation. The arterial load refers to the resistance that the ventricles encounter when pushing blood into the central arterial compartment. In an optimally coupled system, the ventricles effectively match their pumping ability with the arterial load and with a resultant arterial pressure allowing peripheral autoregulation of blood flow throughout the vascular tree. Healthy VAC results in optimal cardiac output, adequate peripheral organ perfusion and an efficient circulation, while an imbalance, referred to as “uncoupling”, can lead to impaired cardiac function and potential cardiovascular failure [1]. Understanding and maintaining VAC is useful for assessing cardiac performance and designing effective treatments for various cardiovascular conditions [8]. VAC can be graphically described using ventricular pressure–volume relations during a cardiac cycle and superimposing the associated arterial pressure to stroke volume relation.

**Fig. 1:**
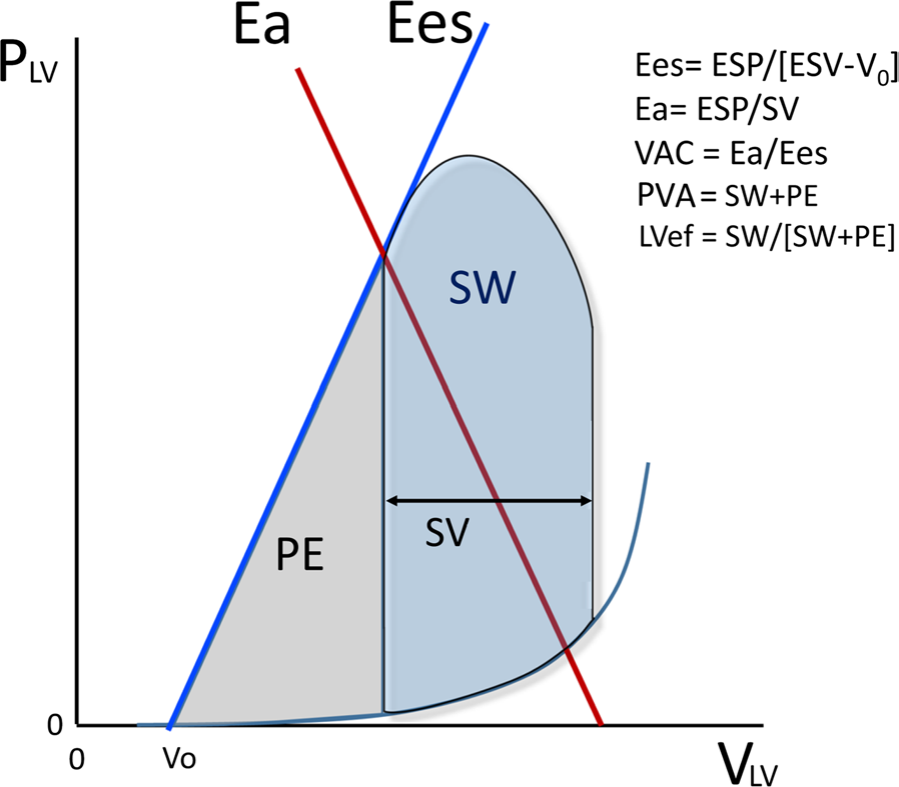
Pressure/volume loop describing ventriculo-arterial coupling. *Plv* left ventricular pressure, *Vlv* left ventricular volume, *ESP* left ventricle end-systolic pressure, *ESV* left ventricle end-systolic volume, *EDV* left ventricle end-diastolic volume, *Ea* arterial elastance, *Ees* end-systolic elastance, *VAC* ventriculo-arterial coupling, *LVeff* left ventricular efficiency, *PVA* pressure/volume area, *SW* stroke work, *PE* potential energy, *SV* stroke volume. Modified from Guarracino et al. (2019) Cardiovascular determinants of resuscitation from sepsis and septic shock. Crit Care 23:118 (http://creativecommons.org/licenses/by/4.0/)

Left ventricular systolic pump function can be simplistically quantified as the end-systolic pressure–volume relations (ESPVR) whose slope, called end-systolic elastance (Ees) (Fig. 1), defines the maximal LV stiffness or elastance at end-systole [5, 6, 9]. Importantly, both the ESPVR and Ees are independent of arterial pressure, though the exact end-systolic volume and this stroke volume created on a single beat are highly dependent on the arterial pressure. As arterial pressure increases for the same Ees, end-systolic volume will also proportionally increase. If LV contractility were to decrease it would manifest itself as a decrease in Ees, whereas an increase in contractility would increase Ees.

Similarly, the arterial outflow pressure can also be described as the relation between changing LV stroke volume and end-systolic pressure (Fig. 1). The slope of the line describing changes in stroke volume to changes in arterial pressure defined the arterial vascular stiffness and is a function of vasomotor tone and the viscoelastic properties of the large arterial vessel walls. In analogy to Ees, it is called arterial elastance (Ea). And just like Ees, if arterial tone were to decrease, Ea would decrease and if arterial tone were to increase Ea would increase. Traditionally one plots Ea on the LV pressure–volume relation as a line with a negative slope starting at zero pressure and maximal end-systolic volume and intersecting the LV pressure–volume loop at end-systole.

**Fig. 2:**
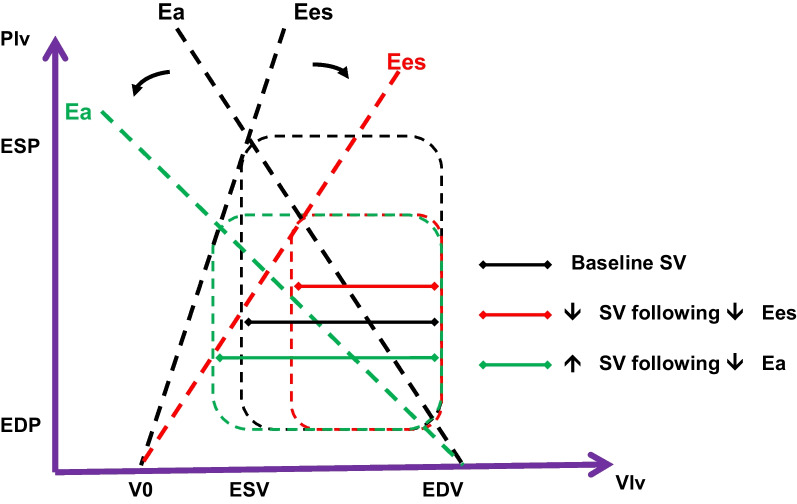
Effect of alterations in either Ees (**A**) or Ea (**B**) on stroke volume. *P* left ventricular pressure, *V* left ventricular volume, *ESP* left ventricle end-systolic pressure, *EDP* left ventricle end-diastolic pressure, *ESV* left ventricle end-systolic volume, *EDV* left ventricle end-diastolic volume, *Ea* arterial elastance, *Ees* end-systolic elastance

The ratio Ees/Ea defines VAC (Fig. 1). Normal VAC values are new unity, ranging between 0.8 and 1.36 under normal circumstances. Decoupling is defined as VAC values outside this range. Selectively decreasing contractility (Fig. 2a) will result in a decrease in end-systolic arterial pressure and an increase in end-systolic volume resulting in a decreased stroke volume. Similarly, a selective decrease in arterial vasomotor tone (Fig. 2b) will also decrease end-systolic arterial pressure but end-systolic volume will decrease causing stroke volume to increase. Thus, for the same decrease in arterial pressure stroke volume can either increase or decrease depending on the primary cause of that change, either ventricular contractility or arterial tone. In practice, both arterial tone and contractility can be depressed in severe sepsis. However, if Ees and Ea are markedly dissimilar, decoupling occurs resulting in impaired LV ejection efficiency. If this decoupling persists, acute heart failure can ensue [1, 8–11]. Importantly, decoupling can be due to changes in Ees, Ea or both.

In disease states where the cardiovascular system is involved either primarily or secondarily to treatments, VAC is frequently altered [1]. Furthermore, as will be discussed below, VAC estimations can be valuable for defining the pathophysiological alteration of cardiovascular function in septic shock and guiding therapeutic interventions [8, 12].

### Bringing VAC physiology to bedside

Patients with septic shock show a large heterogeneity in response to standardized treatment making the effective application of such standardized treatments across patients problematic [13]. Much of the pathophysiological reasons for this heterogeneity of response to treatments is understandable through an analysis of VAC. To illustrate this we describe below three main clinical scenarios that are often encountered in septic shock:

- myocardial depression with preserved cardiac output (CO)
- heterogeneous response to vasopressors in hypotension
- pulmonary artery hypertension in acute respiratory distress syndrome (ARDS)

### Myocardial depression with preserved CO

Myocardial depression is often seen in septic patients, though CO may not be decreased if fluid resuscitation is also given, owing to the associated decrease in arterial pressure caused by sepsis-induced vasoplegia. We have previously described the physiology of VAC in critically ill septic patients [1] and the role of altered VAC in the specific setting of septic shock [14].

In analyzing a cohort of septic shock patients in whom Ea and Ees were measured at several time intervals [3], we noted that most of the septic shock patients show ventriculo-arterial decoupling at the time of septic shock diagnosis and that reduced myocardial contractility is present despite an associated decreased arterial vasomotor tone.

Most clinicians consider that reduced myocardial function should cause reduced systemic flow. However, by analyzing VAC during sepsis, one can see how blood flow can be maintained despite a reduced Ees and a preserved LV ejection fraction (LVEF). Recall that LVEF depends on both contractility and arterial elastance. Vasodilators increase LVEF while vasopressors decrease it. This implies that the use of vasopressors or other agents that selectively increase arterial tone in a septic shock patient with myocardial depression, while potentially improving blood pressure, may decrease LVEF enough to reduce CO. Indeed, the use of nitric oxide synthetase inhibitors in sepsis uniformly demonstrated that the increase in arterial pressure was associated with a decrease in CO [15]. Furthermore, we documented similar CO decreases in some hypotensive patients receiving norepinephrine [3].

**Fig. 3:**
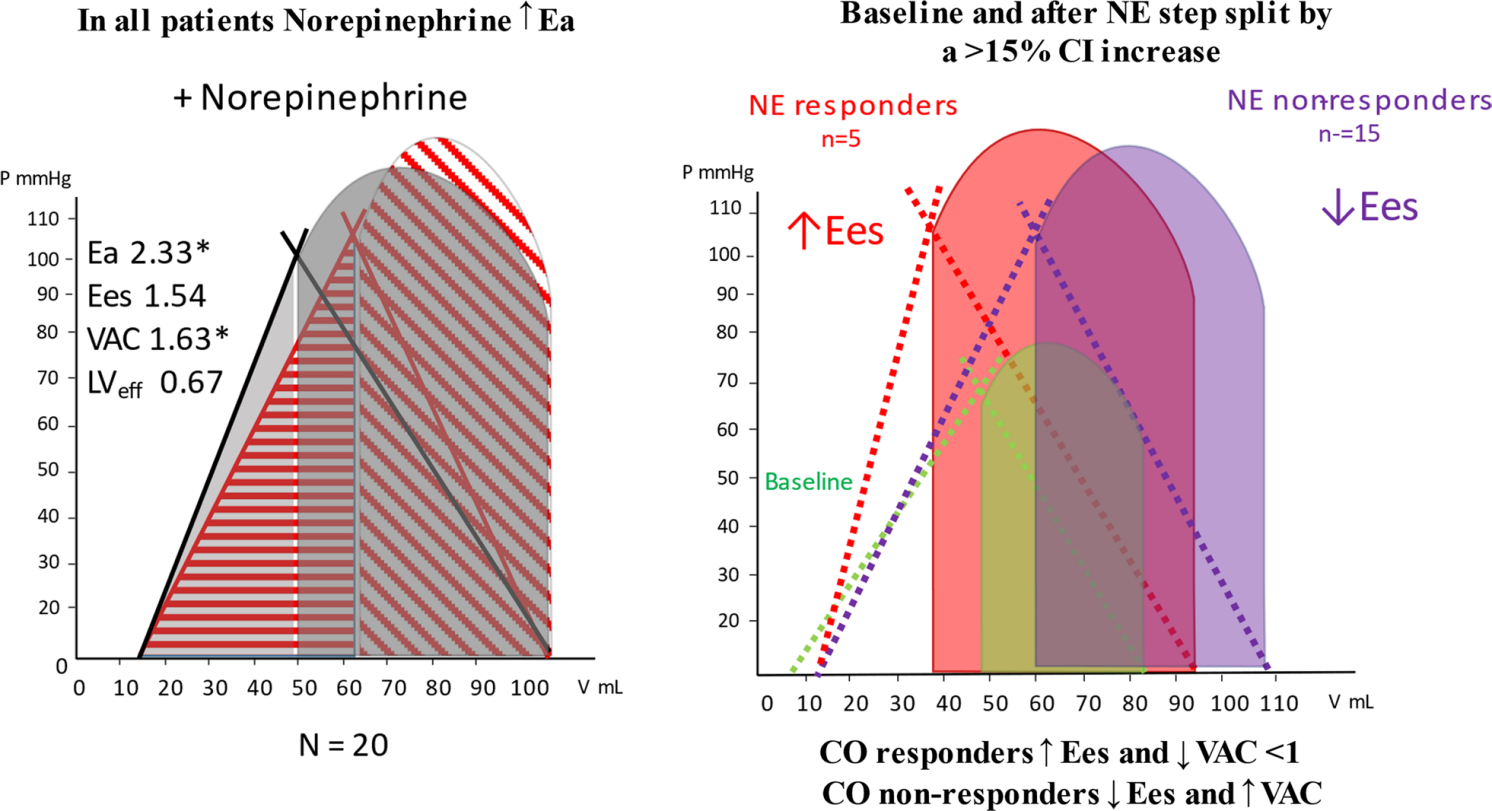
Effect of vasopressor on systolic pressure and stroke volume in the presence of reduced Ees. *P* left ventricular pressure, *V* left ventricular volume, *ESP* left ventricle end-systolic pressure, *EDP* left ventricle end-diastolic pressure, *ESV* left ventricle end-systolic volume, *EDV* left ventricle end-diastolic volume, *Ea* arterial elastance, *Ees* end-systolic elastance, *VAC* ventriculo-arterial coupling, *LVeff* left ventricular efficiency, *CO* cardiac output, *CI* cardiac index, *NE* norepinephrine. Modified from Guarracino et al. (2019) Cardiovascular determinants of resuscitation from sepsis and septic shock. Critical Care 23:118 (http://creativecommons.org/licenses/by/4.0/)

The case of myocardial depression and preserved CO in a septic shock patient following volume resuscitation nicely illustrates how by measuring VAC we can understand whether our treatments can have hemodynamic consequences beyond the apparently responsive (for example preserved CO) or non-responsive (persistent low MAP) macro-hemodynamic values coming from bedside monitoring displays. A decoupled physiology means a high myocardial energetic cost to maintain the “normal values” of CO [1], and explains why, despite preserved CO, blood pressure often does not reach the recommended target threshold values with volume administration alone, and also suggests caution with drugs increasing Ea as it may worsen decoupling in the face of impaired Ees [14] (Fig. 3).

A typical example of such pathophysiological inference on clinical management is described by one of the patients in whom we administered norepinephrine to restore MAP < 65 mmHg despite fluid resuscitation in the presence of preserved CO. In this case, we observed [3] that vasopressor, while increasing MAP, further worsened the VAC, causing left ventricular end-systolic volume to increase and stroke volume to decrease so making the cardiovascular system inefficient and not increasing CO (Fig. 3).

### Heterogeneous response to vasopressors

The current recommended approach to rapidly counteract the impact of sepsis on blood flow in the first few hours after diagnosis and promptly restore adequate blood flow and perfusion pressure consists of an initial volume expansion (VE) with crystalloids to achieve the mean arterial pressure (MAP) of at least 65 mmHg [2]. If this initial treatment fails to restore MAP, then clinicians are allowed to use vasopressors agents and subsequently inotropic support to achieve this goal.

This standardized approach to cardiovascular stabilization achieves the target in restoring MAP in the majority of patients but not in all. Previously reported success rate for VE was around 60%, and we also reported that such approach is not successful in all cases [3], and underlined the heterogeneous response of MAP [13].

From a physiological point of view fluid resuscitation increases circulating blood volume and thus mean systemic pressure (Pms) [16–18], which is the upstream pressure driving venous return and then allowing CO to increase. However, for an increase in Pms to increase CO, the right ventricle needs to be volume responsive as manifest by an associated increase in the right atrial pressure (Pra) to Pms gradient, because venous return can only increase if this gradient increases, the resistance to venous return decreases or both. Finally, in a fluid-responsive septic patient for MAP to also increase in parallel to the increase in CO, arterial tone must be adequate enough to realize an associated increase in pressure to follow the increase in flow as quantified by Ea. Despite the strong rationale of this physiological approach, it is still unclear how these processes play out in individual patients presenting with hypotensive sepsis and why sometimes the response to vasopressors can be disappointing.

We showed in a previous clinical study that NE could increase Ea and MAP in most hypotensive septic patients but did not achieve a MAP > 65 mmHg in a majority and induced ventriculo-arterial uncoupling to levels seen prior to resuscitation [3]. This decreased LV ejection efficiency, if sustained, might impair LV performance. These data support the clinical finding that sustained vasopressors use of > 6 h to maintain a MAP > 75 mmHg in septic shock is associated with increased mortality. We observed that only patients with higher Ees and normalized VAC increased CO during NE infusion, presumably because they can tolerate the increased afterload.

When dobutamine was added to VE and norepinephrine in a few patients, it restored normal VAC and CO, suggesting that inotropic support may improve contractility in septic patients who may be affected by septic cardiomyopathy.

**Fig. 4:**
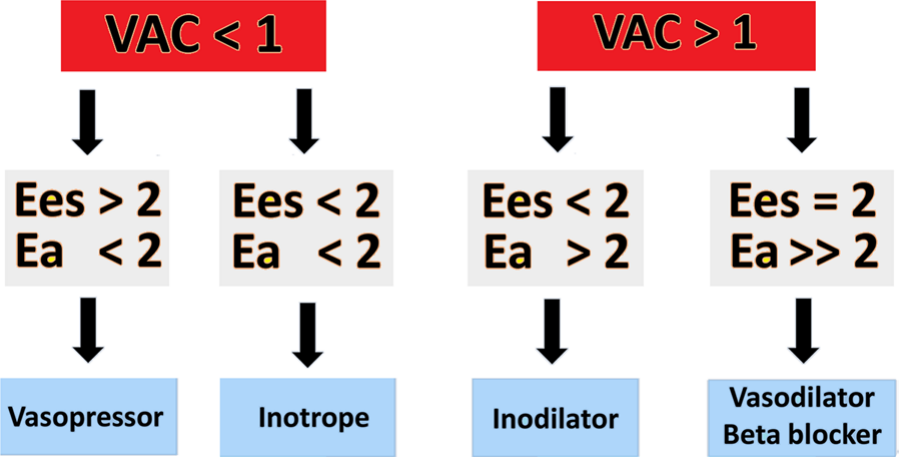
Ventriculo-arterial coupling guided hemodynamic treatment. *Ees* ventricularelastance, *EA* arterialelastance, *VAC* ventriculo-arterialcoupling. (Modified from: Guarracino et al. Management of cardiovascular insufficiency in ICU: the BEAT approach. Minerva Anestesiol 2021 87(4):476–480. With permission.)

These observations support the concept of monitoring VAC at the bedside in septic shock patients to personalize treatment and assess the response. We recently suggested a structured approach that incorporates VAC measurement and hemodynamic monitoring to better inform treatment decisions (FIG BEAT modified) [8]. Based on elastance values and their ratio, one can focus treatment either on the volume side or on the vasopressor and inotrope side. Moreover, in septic patients exhibiting persistent tachycardia even after achieving hemodynamic stabilization, the assessment of VAC can help determine whether introducing a beta-blocker is appropriate to reduce heart rate and improve myocardial efficiency. Specifically, if the evidence shows preserved Ees in the presence of increased Ea (Fig. 4), this supports the decision to administer a beta-blocker without the risk of negative hemodynamic consequences.

### Pulmonary artery hypertension in ARDS and sepsis

Pulmonary hypertension (PulHPT) and right ventricular (RV) dysfunction can have significant implications in sepsis complicated by acute respiratory distress syndrome (ARDS). ARDS occurs in approximately 6–7% of the patients with sepsis [19, 20], whether because of a primary pulmonary process (i.e., pneumonia, aspiration) or secondary one (i.e. peritonitis, pancreatitis). During sepsis, the body’s response to infection triggers widespread inflammation and lead to the development of ARDS, which is characterized by severe respiratory failure. Although most clinicians focus on the effects of ARDS on gas exchange and lung compliance, increased pulmonary Ea can also occur, especially in patients receiving mechanical ventilation and higher levels of positive end-expiratory pressure. Whatever the primary disease, the systemic inflammation can damage the pulmonary vasculature, resulting in increased pulmonary vascular resistance and elevated pulmonary arterial pressures, leading to pulmonary hypertension. As PulHPT worsens, the right ventricle faces increased afterload. This increased workload can lead to right ventricular dysfunction, compromising the heart’s ability to effectively pump blood and maintain cardiac output [21].

In combined sepsis and ARDS, the development of pulmonary hypertension leads to disrupted right-sided VAC [22, 23], making the following pathophysiological picture happen:

Pulmonary hypertension leads to an increase in the afterload on the right ventricle (RV). In the context of sepsis with ARDS and pulmonary hypertension, RV ventriculo-arterial decoupling occurs due to both an increase in pulmonary Ea and a decrease in RV Ees. Thus, the right ventricle’s ability to generate pressure and sustain CO is limited. As RV workload increases further, its contractile function may become impaired, further decreasing RV stroke volume and CO, while increasing right atrial pressure. Such venous congestion further impairs organ blood flow.

Overall, the disruption of RV VAC due to both pulmonary hypertension and right ventricular dysfunction in sepsis with ARDS can have significant consequences on cardiovascular function. Monitoring and managing the hemodynamic status of these patients is essential to optimize ventricular performance and maintain adequate blood flow throughout the body. Interventions may include vasopressors to sustain RV coronary blood flow, inotropic agents, and therapies aimed at reducing pulmonary vascular resistance to improve ventricular–arterial interaction and overall cardiac function [24, 25].

### How to measure VAC at the bedside

Since VAC is defined by the ratio of arterial elastance (Ea) to left ventricle (LV) end-systolic elastance (Ees), the tools used must be capable of measuring both Ea and Ees [23].

Invasive ventricular catheterization allows for pressure/volume loop analysis, which was initially demonstrated by Suga and Sugawa [26–29] can be used to estimate the ESPVR and Ees. However, this approach is not practical for bedside ICU care.

To assess ventriculo-arterial coupling (VAC) in critically ill patients, diagnostic tools that can be easily brought to the bedside and used repeatedly are required. Non-invasive methods can be used. Ea can be estimated as the ratio of end-systolic pressure to stroke volume. End-systolic pressure is approximately 90% of systolic pressure. Ea is calculated as [0.9 × systolic arterial pressure]/SV) [28]. However, for a clinician to use this formula at the bedside heart rate must remain constant, because changes in heart rate will independently alter this calculation even if Ea is unchanged.

Ees can be estimated using the modified single-beat method proposed by Chen et al. It is the most accurate and well-validated to estimate Ees [30–32]. This non-invasive method utilizes echocardiography to estimate Ees from LV end-diastolic and end-systolic areas, along with systolic and diastolic arterial pressure measurements. While the Chen et al. method is considered the clinical reference for non-invasive assessment of VAC, other non-invasive methods are available that estimate Ees, such as the ESP/ESV-based methods. However, these alternative methods may not adequately substitute the Chen et al. method for assessing changes in VAC induced by therapeutic interventions [28].

**Fig. 5:**
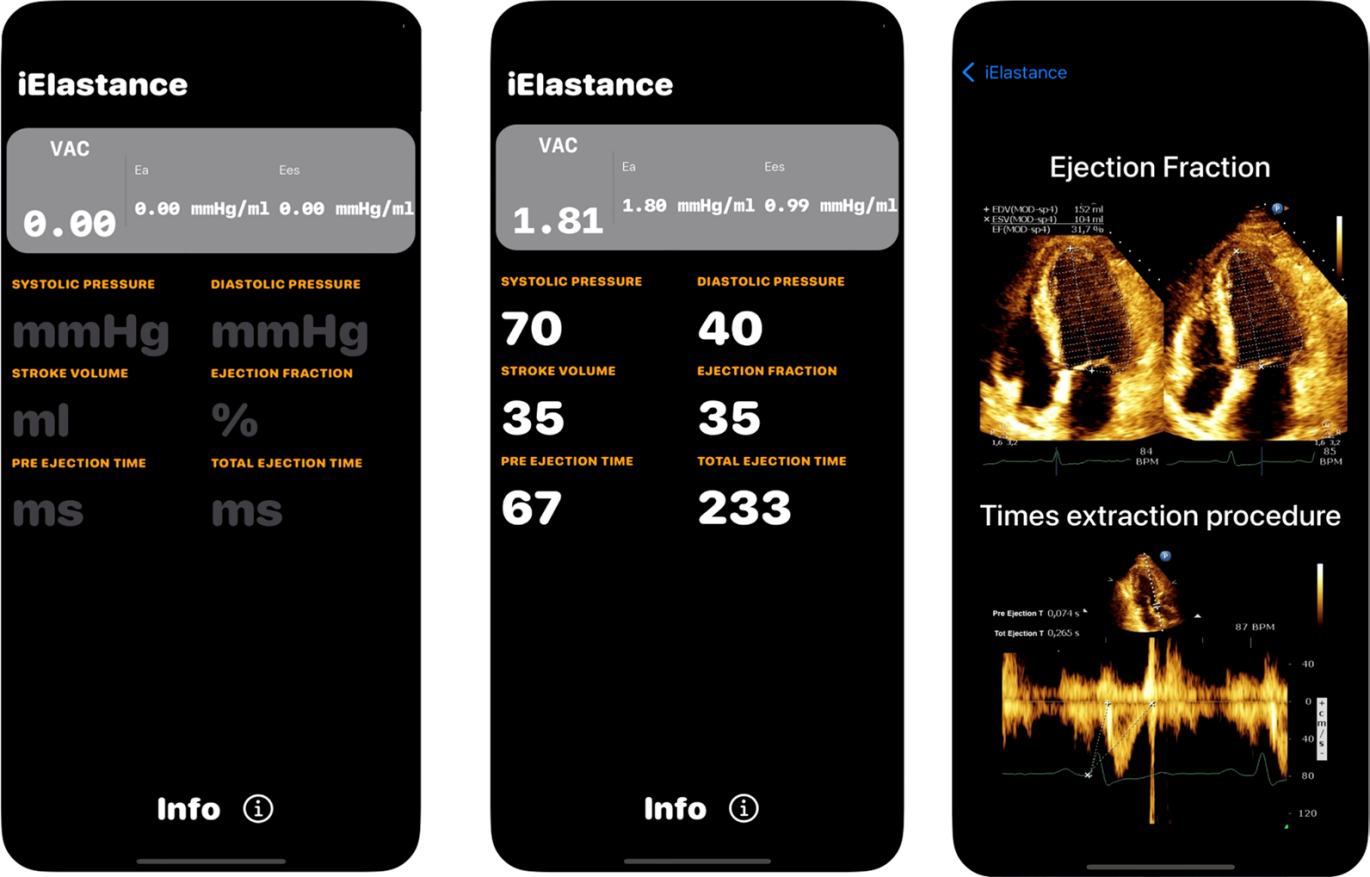
iElastance App. Operator inserted blood pressure, stroke volume, ejection fraction, and ejection times allows for the immediate display of Ea, Ees and VAC. On the right panel the echo images show how to measure the left ventricle ejection fraction (EF) and to detect the systolic ejection times. Abbreviations as defined in the text

To facilitate the bedside calculation of Ees, we developed a mobile app called “iElastance” (Fig. 5), freely available online [33] that calculates VAC using the Chen et al. method. The application utilizes echocardiographic measures (stroke volume, ejection fraction, total ejection time, and pre-ejection time) and hemodynamic parameters (blood diastolic and systolic pressure) to calculate Ea, Ees and subsequently VAC.

The application is easy to use and provides rapid bedside assessment of VAC following a focus echocardiographic examination. While the application cannot replace clinical evaluation and judgement, it can be helpful, especially in critically ill patients where the initial therapeutic intervention did not achieve the expected effect, In the recent literature, the iElastance app has been reported to be easily measurable and have a clinical impact [34, 35].

Measuring the coupling between the right heart ventricle and the arterial system in septic patients is also essential for understanding RV performance and its interaction with the pulmonary circulation, especially in light of the pathophysiological scenarios described above [36].

Similar to the left heart, invasive ventricular and pulmonary artery catheterization allows for pressure/volume loop analysis. However, non-invasive approaches have also been introduced for assessing the right side of the heart [37–39], the majority based on echocardiography. Though several methods have been proposed to noninvasively measure pulmonary artery Ea from pulmonary artery pressure trace and Ees of the RV based on ventricular volume assessment by echocardiography, the most validated measure of right heart VAC is the ratio of tricuspid annular plane systolic excursion (TAPSE) to pulmonary artery pressures (PAPs) [20, 21, 40–44]. This measure, differently from others, can be rapidly obtained at the bedside of a critically ill septic patient through echocardiography without the challenges of right ventricular volume measurement.

## Conclusions

The considerable variability observed in responses to time and treatment of septic shock patients poses challenges when trying to apply standardized interventional protocols. The inherently varied VAC status of these patients affecting Ees, Ea or both requires that the bedside clinician develop a deeper understanding of the mechanisms underlying the hemodynamic instability and to measure VAC in complex patients. Non-invasive echocardiographic assessment of VAC has shown to be a valuable tool in evaluating the intrinsic factors contributing to hemodynamic impairment in human septic shock and monitoring the effectiveness of therapeutic interventions (Table 1).

**Table 1:** Effects of vasopressors and inotropes on elastances and ventriculo-arterial coupling

	Ees	Ea	VAC (Ea/Ees)
Norepinephrine	= /↑	↑	= /↑
Epinephrine	↑	↑/↓	= /↓
Vasopressin	=	↑	↑
Angiotensin II	=	↑	↑
Dobutamine	↑	↓	↓
Milrinone	↑	↓	↓
Levosimendan	↑	↓	↓

*Ees* ventricular elastance, *Ea* arterial elastance, *VAC* ventriculo-arterial coupling

^*^Depending on the given dose, different alpha and beta receptors are stimulated

Given that septic shock patients often have impaired cardiovascular reserve, a more personalized approach to therapy, tailored based on volume responsiveness and VA coupling, may be essential to achieve effective and efficient resuscitation from severe sepsis. Integrating bedside assessment of Ea and Ees with dynamic indexes of fluid responsiveness provides a multi-modality approach that enhances our understanding of the pathophysiology and should guide personalized management of the severe hemodynamic instability seen in septic shock.

## Data Availability

Not applicable.
